# Donohue syndrome with a homozygous INSR exon 14 deletion: a case report

**DOI:** 10.1093/omcr/omaf183

**Published:** 2025-09-28

**Authors:** José Daniel Almazán Monroy, Arodys Julianny Valle Martinez, Sara Elizabeth Milla Salguero, Eduardo Smelin Perdomo Domínguez

**Affiliations:** Department of Pediatrics, Hospital Nacional Mario Catarino Rivas, 1st Street, Barrio El Playón, San Pedro Sula, 21102, Honduras; Instituto Hondureño de Seguridad Social, Boulevard del Norte, San Pedro Sula, 21102, Honduras; Instituto Hondureño de Seguridad Social, Boulevard del Norte, San Pedro Sula, 21102, Honduras; Department of Internal Medicine, Fundación Ruth Paz, 7th-8th Street, 9th Avenue, Barrio Lempira, San Pedro Sula, 21104, Honduras; Médico General, Hospital Nacional Mario Catarino Rivas, 1st Street, Barrio El Playón, San Pedro Sula, 21102, Honduras; GIMUNICAH, Faculty of Medicine, Universidad Católica de Honduras, Colonia Zerón, 12th Street, 21st Avenue, San Pedro Sula, 21101, Honduras

**Keywords:** Donohue syndrome, insulin, insulin resistance, INSR, IGF-1

## Abstract

Donohue syndrome (DS) is a rare autosomal recessive disorder caused by homozygous or compound heterozygous mutations in insulin receptor (INSR), leading to severe insulin resistance. It is characterized by extreme hyperinsulinemia, fasting hypoglycemia, postprandial hyperglycemia, severe growth restriction, and dysmorphic features, with a high mortality rate in the first year due to metabolic instability and infections. We report the case of a 3-month-old Honduran girl with a homozygous exon 14 deletion in INSR who presented with severe insulin resistance, metabolic dysregulation, and dysmorphic facial features. Despite treatment with octreotide, metformin, and maltodextrin, the patient’s condition worsened, leading to septic shock, disseminated intravascular coagulation, and multiple organ failure. This case highlights the challenges in correlating genotype with phenotype in DS and emphasizes the importance of understanding how specific INSR mutations influence the treatment response and clinical outcomes.

## Introduction

Donohue syndrome (DS; #OMIM 246200) is a rare autosomal recessive disorder caused by homozygous or compound heterozygous mutations in insulin receptor (INSR) gene on chromosome 19p13, leading to defective insulin receptor function. It is an extremely rare condition, with an estimated prevalence of less than 1 case per million births [1]. Most cases are reported in the context of parental consanguinity due to the autosomal recessive inheritance; however, consanguinity is not mandatory, and sporadic cases in non-consanguineous families have been described [2].

DS is characterized by severe hyperinsulinemia, fasting hypoglycemia, postprandial hyperglycemia, growth restriction, and dysmorphic features, with most patients dying within the first year of life due to metabolic instability and infections [3]. Recombinant human IGF-1 (rhIGF-1) has been explored as a potential therapy for bypassing insulin receptor dysfunction. Here, we describe a patient with a homozygous exon 14 deletion in INSR born to non-consanguineous parents. To our knowledge, this is the first documented case of DS in Honduras, highlighting the challenges of genotype–phenotype correlation and the need to better understand how treatment response and clinical outcomes correlate with specific mutations.

## Case report

We report the case of a Honduran 3-month-old girl born to a 25-year-old female. She was the second child of non-consanguineous parents. Pregnancy was complicated by intrauterine growth restriction, resulting in preterm delivery at 36 weeks of gestation. She was delivered via cesarean section due to a previous cesarean delivery. Birth weight was 1600 g, length 46 cm, head circumference 31 cm, with Apgar scores of 8 and 9.

**Figure 1:**
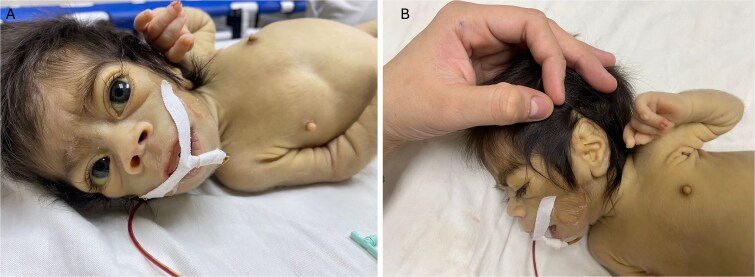
Dysmorphic facial features in Donohue syndrome. (A) Frontal view showing prominent eyes, hypertelorism, hypertrichosis, a short, upturned nose, a wide mouth, and scleral icterus. (B) Lateral view highlighting elongated low-set ears with prominent lobes and micrognathia.

**Figure 2:**
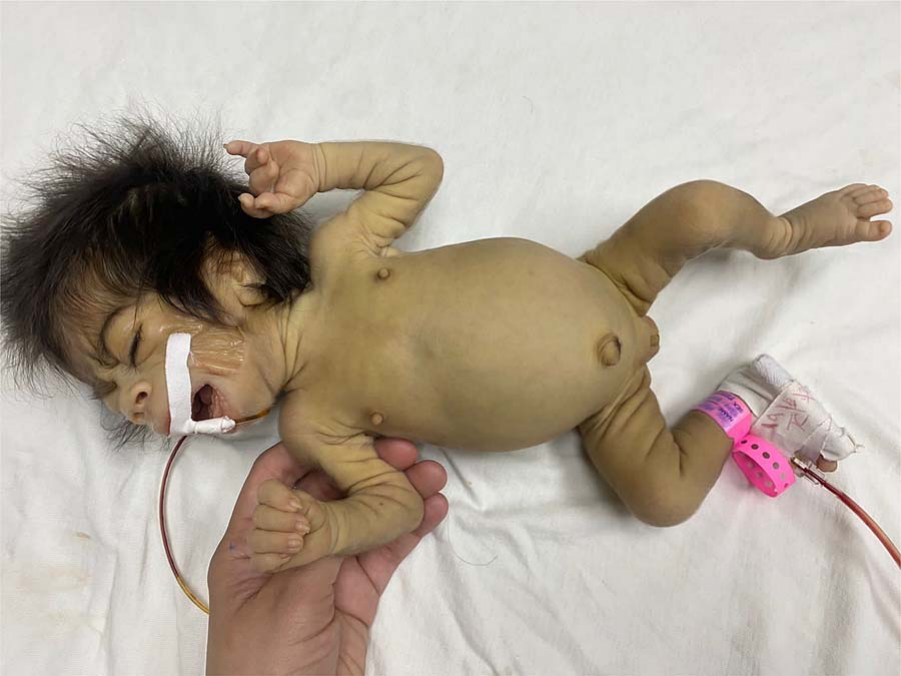
Clinical features of Donohue syndrome. Generalized lipoatrophy, prominent nipples, and disproportionately large hands and feet are observed.

On initial physical examination, she presented with dysmorphic facial features, including prominent eyes, hypertelorism, hypertrichosis, a short, upturned nose, wide mouth, elongated low-set ears with prominent lobes, high forehead, micrognathia, and high palate (Fig. 1A and B). She also exhibited double major scoliosis, stiff skin, lipoatrophy, prominent nipples, and disproportionately large hands and feet (Fig. 2). Genitalia were normal. Additionally, hepatomegaly was noted, with the liver edge palpable 4 cm below the right costal margin.

Immediately after birth, she was admitted to the NICU because of low birth weight, respiratory distress, fever, jaundice, and early-onset sepsis, treated empirically with antibiotics.

During hospitalization, she developed severe metabolic instability (hypoglycemia 18 mg/dL, postprandial hyperglycemia 200 mg/dL) requiring IV dextrose infusion. She also presented with persistent vomiting and abdominal distension, raising suspicion of necrotizing enterocolitis despite formula adjustment.

Laboratory analysis revealed insulin 990.4 ng/ml, C-peptide 9.74 ng/ml, IGF-1 7.0 ng/ml, and cholestatic jaundice with elevated direct bilirubin. Additional laboratory findings are summarized in Table 1. Abdominal ultrasound was otherwise unremarkable, except for the presence of free fluid within the abdominal cavity.

**Table 1:** Patient’s biochemical parameters and corresponding reference values.

Parameter	Patient Value	Reference Range
Fasting glucose	18 mg/dl	70–100 mg/dl
Postprandial glucose	200 mg/dl	<140 mg/dl
Insulin	990.4 ng/ml	0.09–0.86 ng/Ml
C-peptide	9.74 Ng/Ml	0.8–3.1 ng/ml
IGF-1	7.0 ng/ml	17–185 ng/ml
TSH	0.58 μIU/ml	0.72–11.0 μIU/ml
Free T4	1.18 ng/dl	0.83–3.09 ng/dl
Estradiol	222 pg/ml	<25 pg/ml
Cortisol	>50.0 μg/dl	6–23 μg/dl
Total Bilirubin	16.9 mg/dl	0.05–0.68 mg/dl
Direct Bilirubin	10.4 mg/dl	0.05–0.30 mg/dl
Potassium	2.1 mEq/l	3.0–6.0 mEq/l
Uric acid	0.30 mg/dl	1.6–6.3 mg/dl
Total Cholesterol	57.40 mg/dl	>70–<170 mg/dl
WBC	32 990/μl	5000–19 500/μl
Platelets	25 000/μl	180 000–327 000/μl

Biochemical values obtained during hospitalization with corresponding age- and sex-specific reference values. IGF-1, insulin-like growth factor 1; TSH, thyroid-stimulating hormone; WBC, white blood cell count.

The combination of severe insulin resistance, dysmorphic facial features, and multiple metabolic abnormalities raised suspicion of an INSR-related disorder. Genetic analysis confirmed a pathogenic homozygous deletion of exon 14 in the INSR gene, supporting the diagnosis of DS. Parental genetic testing was not performed.

The patient was treated with octreotide, metformin, and maltodextrin. However, she experienced progressive clinical deterioration, and despite intensive supportive care, developed septic shock complicated by disseminated intravascular coagulation, which led to severe gastrointestinal bleeding, culminating in multiple organ failure and death.

## Discussion

DS is a rare autosomal recessive disorder linked to INSR mutations [3]. Most pathogenic variants are missense (64%) or nonsense (13%) mutations, while deletions account for approximately 8.3% of reported cases [4]. We describe the first reported case of DS in Honduras, involving a patient with a homozygous exon 14 deletion presenting with the classic metabolic features of DS.

In severe insulin resistance syndromes, INSR loss-of-function mutations play a central role in disease severity. Mutations affecting the α-subunit (exons 1–11) are often associated with more severe phenotypes, but disruptions in the β-subunit (exons 12–22) can also significantly impair insulin signaling [4]. The exon 14 deletion in our patient led to a frameshift mutation and premature termination codon, resulting in defective receptor expression due to reduced mRNA levels [5].

Despite exhibiting classic features of DS, our patient experienced early mortality at three months. In contrast, a previously reported case with a complete homozygous INSR deletion survived until 15 months under intensive nutritional support, including frequent daytime feedings and continuous enteral nutrition [6]. This disparity highlights that survival in DS may be influenced not only by the type of mutation but also by the clinical setting, access to supportive therapies, and potential compensatory mechanisms.

Evidence from animal models indicates that IGF-1R signaling provides a degree of compensation, since IGF-1R deletion is lethal whereas INSR loss is not [6]. This has led to the use of rhIGF-1 as a potential therapy to bypass defective insulin signaling. Case reports suggest that sustained rhIGF-1 infusion may improve glycemic control, liver function, and cardiac hypertrophy [7].

Long-term studies suggest that patients with heterozygous or hypomorphic INSR mutations, retaining partial receptor function, show improved survival [8]. Moreover, early identification and management of life-threatening comorbidities such as hypertrophic cardiomyopathy or pancreatic exocrine insufficiency may positively affect outcomes [9, 10]. In addition, early initiation of rhIGF-1 therapy has demonstrated both acute and chronic metabolic benefits and remains the only treatment with documented efficacy in some cases of DS. Although no guidelines exist for its use, a clinical protocol developed by the Department of Pediatrics in Cambridge recommends starting rhIGF-1 at low doses with gradual escalation to 0.4 mg/kg/day [11].

Our patient was managed with octreotide, metformin, and maltodextrin for metabolic control. However, despite these interventions, progressive clinical deterioration was observed, including worsening abdominal distension, metabolic decompensation, and eventual progression to septic shock with multiorgan failure, culminating in early mortality. Additionally, the treatment response may be influenced by the type of INSR mutation, as demonstrated in a patient with a compound heterozygous mutation who achieved glycemic stability with metformin and continuous nasogastric feeding [12].

This report emphasizes the limitations of available therapies, particularly in low-resource settings where rhIGF-1 is unavailable. Although functional studies were not feasible, our findings highlight the need for mutation-specific therapeutic insights and improved access to effective treatments.

## References

[1] Nijim Y, Awni Y, Adawi A. et al. Classic case report of Donohue syndrome (leprechaunism; OMIM 246200). Medicine (United States) 2016;95:e2710. 10.1097/MD.0000000000002710 PMC4753905 26871809

[2] Zhou Q, Yu J, Yuan X. et al. Clinical and functional characterization of novel INSR variants in two families with severe insulin resistance syndrome. Front Endocrinol (Lausanne) 2021;12:606964. 10.3389/fendo.2021.606964 33995269 PMC8117416

[3] Kirkwood A, Stuart G, Harding L. Donohue syndrome: a review of literature, case series, and anesthetic considerations. Pediatr Anesth 2018;28:23–7. 10.1111/PAN.13273 29148123

[4] Ardon O, Procter M, Tvrdik T. et al. Sequencing analysis of insulin receptor defects and detection of two novel mutations in INSR gene. Mol Genet Metab Rep 2014;1:71–84. 10.1016/J.YMGMR.2013.12.006 27896077 PMC5121292

[5] De Geest N, Bonten E, Mann L. et al. Genotype-phenotype correlation in inherited severe insulin resistance. Hum Mol Genet 2002;11:1465–75. 10.1093/HMG/11.12.1465 12023989

[6] Wertheimer E, Lu SP, Backeljauw PF. et al. Homozygous deletion of the human insulin receptor gene results in leprechaunism. Nat Genet 1993;5:71–3. 10.1038/ng0993-71 7693131

[7] Melikyan MA, Ivannikova TE, Milovanova NV. et al. Donohue syndrome and use of continuous subcutaneous IGF1 pump therapy. Probl Endokrinol (Mosk) 2022;68:79–86. 10.14341/PROBL13121 36337021 PMC9762435

[8] Musso C, Cochran E, Moran SA. et al. Clinical course of genetic diseases of the insulin receptor (type a and Rabson-Mendenhall syndromes): a 30-year prospective. Medicine 2004;83:209–22. 10.1097/01.MD.0000133625.73570.54 15232309

[9] Hovnik T, Bratanič N, Podkrajšek KT. et al. Severe progressive obstructive cardiomyopathy and renal tubular dysfunction in Donohue syndrome with decreased insulin receptor autophosphorylation due to a novel INSR mutation. Eur J Pediatr 2013;172:1125–9. 10.1007/s00431-012-1901-7 23229189

[10] Kostopoulou E, Shah P, Ahmad N. et al. Gastrointestinal dysmotility and pancreatic insufficiency in 2 siblings with Donohue syndrome. Pediatr Diabetes 2017;18:839–43. 10.1111/PEDI.12483 28004474

[11] Semple RK, Williams RM, Dunger DB. What is the best management strategy for patients with severe insulin resistance? Clin Endocrinol 2010;73:286–90. 10.1111/J.1365-2265.2010.03810.X 20455892

[12] Kirel B, Bozdağ Ö, Köşger P. et al. A case of donohue syndrome “leprechaunism” with a novel mutation in the insulin receptor gene. Turk Pediatri Ars 2017;52:226–30. 10.5152/TURKPEDIATRIARS.2017.3193 29483803 PMC5819861

